# The Practical Application of Bio-Inspired PMA for the Detection of Partial Discharges in High Voltage Equipment

**DOI:** 10.3390/s23239307

**Published:** 2023-11-21

**Authors:** Josiel Cruz, Alexandre Serres, Raimundo Freire, Edson Costa, George Xavier, Adriano Oliveira, Vladimir Souza, Pavlos Lazaridis

**Affiliations:** 1 Post-Graduate Program in Electrical Engineering (PPgEE), Coordination of Postgraduate Studies in Electrical Engineering, Federal University of Campina Grande (UFCG), Campina Grande 58429-900, Brazil; 2 Electrical Engineering Department, Federal University of Campina Grande (UFCG), Campina Grande 58429-900, Brazilalexandreserres@dee.ufcg.edu.br (R.F.);; 3 Electrical Engineering Department, Federal University of Sergipe (UFS), Sergipe 49100-000, Brazil; 4 Electrical Engineering Department, Federal Rural University of the Semi-Arid (UFERSA), Mossoró 59625-900, Brazil; 5 School of Computing and Engineering, University of Huddersfield, Huddersfield HD1 3DH, UK; p.lazaridis@hud.ac.uk

**Keywords:** bio-inspired design, partial discharges, UHF sensors, monitoring, artificial intelligence

## Abstract

In this paper, the practical application of a bio-inspired antenna for partial discharge (PD) detection in high voltage equipment was evaluated in order to validate the efficiency of using this technology for PD monitoring purposes. For this, PD measurements using the bio-inspired antenna were performed on operational 69 kV potential transformers (PT) in a real substation. After the field experiment, laboratory measurements using the IEC 60270 standard method and a bio-inspired antenna were performed, simultaneously, over the evaluated PT. The results obtained at the substation indicated suspicious frequencies of partial discharge activity in two out of three evaluated potential transformers, mainly for the frequencies of 461 MHz, 1366 MHz, 1550 MHz and 1960 MHz. During the laboratory tests, the presence of partial discharge activity over the suspicious potential transformers was confirmed with the detection of PD apparent charge levels above 20 pC. Finally, the frequency spectrum obtained from the PD signals detected by the bio-inspired antenna in the laboratory presented similar frequency values to those obtained during the practical application at the substation, making it a promising indicator for future defect classification studies using artificial intelligence.

## 1. Introduction

Interruptions often occur due to failures in the insulation systems of high-voltage (HV) equipment in substations, and can be minimized through the use of predictive monitoring methodologies that gather historical information about the occurrence of defects [1] and optimize decision-making processes regarding maintenance and hence asset management in power supply companies.

Energy companies therefore need to improve their maintenance processes, and to adopt procedures that anticipate failures and interruptions. In an electrical system, the monitoring of the insulation system degradation processes in HV equipment is based on the identification of physical, chemical or electrical phenomena that may arise during its operation. The degradation of electrical insulation is characterized by irreversible changes in the internal properties of a dielectric material due to one or more factors such as the existence of non-uniform electric fields distributed over the dielectric material, often originating from loose connections or geometries that favor the concentration of these fields in specific areas of the dielectric, resulting in localized discharges that slowly degrade the insulating material until a dielectric breakdown and consequent equipment failure occurs, thus reducing its lifecycle [2]. These localized discharges are referred to as partial discharges (PDs) and are considered one of the main symptoms and sources of HV insulating system degradation [3,4].

Therefore, the continuous monitoring of PD activity in high-voltage equipment is crucial to prevent the development of HV insulating system problems and consequent equipment failures, resulting in the development of several methods that can be used to verify the occurrence of PDs in HV equipment, such as dissolved gas analysis (DGA) [5], acoustic methods [6], electrical methods (standardized based on IEC 60270) [7] and radiometric methods [8]. DGA is based on an analysis of the content of gases dissolved in oil, and is characterized by its low sensitivity in detecting the onset of PD activity and difficulties in performing continuous and non-invasive monitoring [9].

Although widely used, the acoustic method has certain limitations in terms of identifying PD signals due to phenomena such as reflections, refractions, mechanical noise and attenuation [6]. The electrical method is the most traditional [7], but requires the use of a coupling capacitor connected in parallel to the equipment under analysis, meaning that this method is invasive and subject to noise and interference, and is therefore more suitable for laboratory tests.

The radiometric method is based on the detection of short electromagnetic pulses emitted during the occurrence of PD, generally in the ultra-high frequency (UHF) range (300 MHz up to 3 GHz) [10]. It can be characterized as minimally invasive, since an electrical connection is not required and small sensors or antennas can be used for this purpose [11]. In addition, the radiometric method allows for the monitoring of HV equipment in any HV energized plant [12] by positioning the antennas externally [12] or internally (dielectric windows) [13,14] to the equipment. Moreover, due to its range of operating frequencies, the radiometric method is immune to low-frequency and corona interference [15]. Finally, the radiometric method can provide a more efficient diagnosis of the equipment, thus enabling the supply of information on the detection, classification and location of defects [16].

The classification based on the radiometric method can be carried out using artificial intelligence techniques that identify specific signatures for each of the defects that affect the insulation system of various high voltage equipment, such as: power transformers, current and potential; surge arresters; insulators and others [17]. Among these defects, we can cite internal discharges in insulating systems (solid, liquid or gaseous) or discharges on the boundaries (surfaces) between the various dielectrics that comprise the insulation system of high voltage equipment. Each of these defects will present unique features such as frequency range of occurrence, kurtosis, phase, asymmetry and other statistical parameters [18]. In this way, given the vast number of possible defects in the insulating systems in the most diverse high-voltage equipment, it is necessary to use artificial intelligence techniques, as presented in [19], for their correct classification. However, in order to properly extract the best features, it is crucial for the sensor that is acquiring the PD signals to be as sensitive as possible to the detection of these discharges, robust to background noise and cover the entire spectrum of occurrence of the observed phenomenon.

Hence, in the radiometric method, the use of an antenna that fully covers the main spectrum of PD activity is essential to avoid the detection of false negatives and positives, since the energy of the PD may be more concentrated at specific sub-bands inside the main spectrum of PD activity (300–1500 MHz) [20] according to the type of defect in the insulating system [15]. Therefore, among the various type of antennas that can be used in the radiometric method, the printed monopole antenna (PMA) with ultra-wide bandwidth (UWB) stands out due to its potential detection of PD within the frequency range where the greatest activity of PD occurs. In addition, the use of a PMA is also motivated by its low cost, ease of manufacture and installation, compact size and omnidirectional radiation pattern [21]. Finally, PMAs with bio-inspired geometries have attracted interest for practical applications, including for PD monitoring [14,22,23]. The use of this type of geometry can contribute to the miniaturization process of an antenna without compromising its main parameters [22], and hence optimize the installation of monitoring systems in substations. In addition, bio-inspired geometries provide a higher density of current concentrations on the antenna transmission line, resulting in better gain than classical and modified PMA geometries developed for PD detection, resulting in an antenna with a higher PD detection sensitivity. However, although they have great potential for their application in PD monitoring, the use of bio-inspired PMAs in practice, in a real substation, has rarely been explored in the literature and is limited to superficial investigations presented in [14].

Thus, due to the great practical potential presented by PMAs with bio-inspired geometries and their uncommon use in the literature, this work aims to validate the practical application of the PMA bio-inspired by the leaves of the Inga Marginata plant. This PMA geometry was developed in a previous work [22], in which the built antenna presented very attractive parameters for PD detection applications, such as wide bandwidth (340 MHz–8 GHz), small size (34 cm × 14 cm), average gain of 3.61 dBi, immunity against the detection of corona discharges, good sensitivity for PD detection (65 pC according to the IEC 60270 method) and an omnidirectional pattern (which favors its application in locating PD sources in open environments) [22]. However, all of the aforementioned results and other analyses and tests described in [22] were obtained/performed only in a laboratorial environment. In the laboratory, only an oil cell was used to emulate the occurrence of PD, and it was not possible to verify its applicability in actual high-voltage equipment. In addition, in [22], we had a laboratorial environment without the common obstacles of a substation (various items of high-voltage equipment) and quite controlled, that is, isolated from interferences that are common in substations, such as corona discharges and PD from other equipment. Finally, in the laboratory it was possible to position the antenna closer to the object of analysis (the oil cell), unlike substations in which, for operational safety reasons, the antennas must be positioned at a greater distance. Thus, it is necessary to validate these parameters in practice in a substation operating in real time to verify the PMA’s applicability in detecting PD and, consequently, its future integration with commercial PD measurement systems that use the radiometric method for detection and classification of PD through the application of artificial intelligence. For this purpose, the bio-inspired PMA developed in [22] was used for the measurement of PDs in a 69 kV potential transformer (PT) operating in an actual substation. In addition, in order to validate that the PD pulses detected by the bio-inspired PMA were actually originated in the PT insulating system, laboratory measurements using the IEC 60270 standard method and the bio-inspired PMA were performed simultaneously for the evaluated PT.

## 2. Printed Monopole Antenna

**Figure 1:**
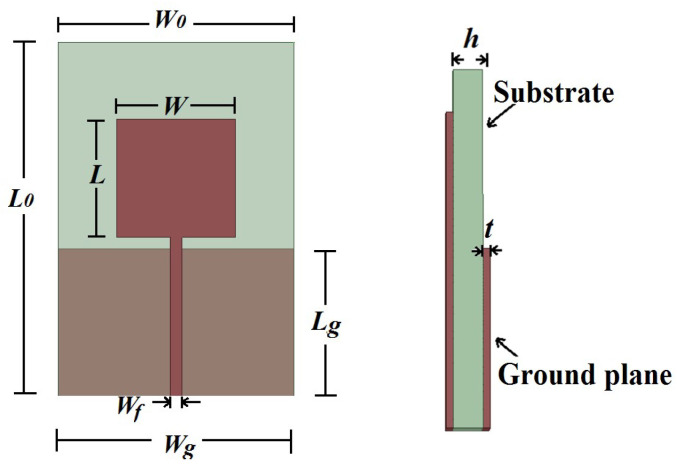
Geometry of a rectangular printed monopole antenna.

Figure 1 shows the basic geometry of a PMA with a rectangular patch, where *W* and *L* are the width and height of the radiating patch, respectively, *W_g_* and *L_g_* are the width and length of the ground plane, respectively, *W_f_* is the width of the transmission line, *W*_0_ and *L*_0_ are the width and height of the antenna, respectively, and *h* is the thickness of the dielectric substrate.

The first frequency (in GHz) of the operating band of the PMA can be approximated by Equation (1), where *p* is the perimeter (in mm) of the antenna patch and *ε_ref_* is the effective relative permittivity (dimensionless) of the dielectric [24].

(1) $$f\left(GHz\right)=\frac{300}{p\sqrt{\varepsilon_{ref}}}$$

$$\varepsilon_{ref}=\frac{\left(\varepsilon_{r}+1\right)}{2}+\frac{\left(\varepsilon_{r}-1\right)}{2}\cdot {\left(1+12\cdot \frac{h}{W}\right)}^{-1/2}$$

where *h/W* > 1, and *h* represents the dielectric thickness (in mm), *W* the microstrip width (in mm) and *ε_r_* the relative permittivity (dimensionless).

According to Ref. [24], the distribution of the electric current density in a PMA is concentrated mainly at the edges of the radiating patch. Thus, increasing the perimeter p increases the wavelength, which in turn decreases the lower operating frequency. Antenna models with bio-inspired patches achieve good miniaturization results, as they have a larger perimeter than conventional antennas [14,22,25,26].

## 3. Methodology

The methodology applied in this research was divided into four different stages. The first two stages were described in detail in [22], and consisted of the design and development of a bio-inspired PMA and laboratory experiments to check the sensitivity of the antenna in terms of PD detection. After obtaining the promising results reported in [22], two additional stages were applied to these antennas to monitor PDs in substations, and the results are presented in this paper. The third stage represents the field application of the bio-inspired PMA to the detection of PDs in a 69 kV PT in the Mussuré II substation (São Francisco Hydroelectric Company, CHESF, São José do Belmonte, Brazil). This substation was chosen due to the presence of equipment suspected of PDs, since other equipment of the same model from the same manufacturer has previously been found to be faulty. In the fourth stage of the applied methodology, the potential transformers evaluated in the substation were collected for a second series of measurements in the laboratory, in which the bio-inspired PMA and the IEC 60270 standard method were applied simultaneously.

### 3.1. Bio-Inspired Antenna Based on the Leaf of Inga Marginata for Detection of PD

**Figure 2:**
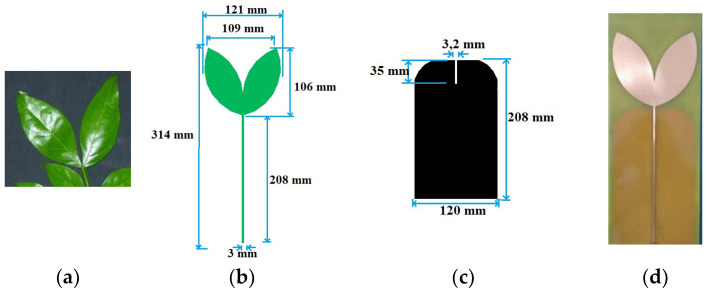
Designed model for the Inga Marginata bio-inspired PMA: (**a**) Inga Marginata leaves; (**b**) patch dimensions; (**c**) ground plane dimensions and (**d**) manufactured bio-inspired antenna [22].

Taking into consideration the requirements of an antenna for an application related to PD detection—such as an operating bandwidth between 300 MHz and 1500 MHz, an omnidirectional radiation pattern and a gain greater than the recommended average limit in order to capture the PD signals (2 dBi) [23]—a low-cost antenna was designed in [22], with a radiating patch inspired by the leaves of the Inga Marginata plant. This bio-inspired shape for the radiating patch was used to reduce the dimensions of the antenna while maintaining its operation in the UHF band. The leaves used as the model, and the dimensions and a photograph of the manufactured antenna, are shown in Figure 2.

More detailed information about the performance (in terms of bandwidth and gain results) of the bio-inspired PMA based on the leaves of the Inga Marginata plant and the results of sensitivity tests for PD detection can be found in previous work [22]. In the following section, we discuss the application of this bio-inspired PMA in the detection of PD in open substations.

### 3.2. Application of the Bio-Inspired PMA in a Substation for PD Detection

In order to evaluate the viability of our bio-inspired PMA under operating conditions, PD measurements were performed at the Mussuré II substation. The equipment evaluated was a set of 69 kV potential transformers, identified as PT 82T3 phase A, phase B and phase C, that were energized and in operation during the measurements. The following instruments were used to take measurements at the substation: a portable spectrum analyzer (model N992A from Agilent Technologies, Santa Clara, CA, USA) to obtain the frequency spectrum in which the PD pulses occurred; a bio-inspired PMA based on Inga Marginata leaves; and a directional antenna (HyperLog 30100X, Latur, India) with an operating band from 380 MHz up to 10 GHz and mean gain equal to 4.55 dBi (without preamplifier) for the frequency range regarding the main activity of PD (300 MHz up to 1500 MHz). This commercial antenna was used for comparison purposes and for the confirmation of PD detected by the bio-inspired PMA.

The procedure adopted for PD identification consisted of an analysis in the frequency domain, which was subdivided into two parts for each of the applied antennas (the bio-inspired PMA and the commercial antenna, a HyperLog 30100X). The first part consisted of identifying the frequency spectrum corresponding to the background noise in the vicinity of the evaluated equipment. For this purpose, the antennas were positioned at a considerable distance from the evaluated PTs (approximately 20 m) and in a region of the substation in which there was, basically, the presence of only overhead conductors and insulators, also located at a significant distance (a height of approximately 6 m). Even though these overhead insulators and conductors present PD activity, such as corona and superficial discharges, the radiated signals for these types of defects present higher energy concentrations for frequencies up to 200 MHz [15]. Therefore, since the antenna has an operating range from 300 MHz up to 1500 MHz, these discharges do not influence the background noise extraction process. Thus, background noise is composed of signals from known sources, such as digital TV signals and mobile phone transmissions.

**Figure 3:**
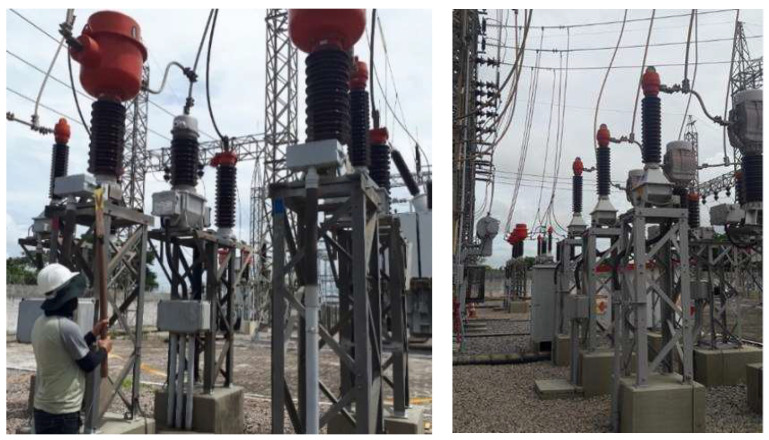
Application of a bio-inspired PMA in the detection of partial discharges in the potential transformers of Mussuré substation.

In this way, it was possible to register the background noise of the substation in general, that is, without major influences from any equipment in the vicinity of the antennas. The background noise extraction stage is an essential step for the identification of PD in the frequency domain, since any changes in the reference spectrum (background noise) when approaching a specific piece of equipment may indicate the possible activity of PD in this equipment. For the acquisition of background noise in the frequency domain, the antennas were connected to the spectrum analyzer, which was configured in “Hold” mode to record the maximum values of frequency signals over a 10 min duration. This time interval proved sufficient for the acquired values to stabilize, resulting in minimal variations in the background noise. This methodology enables the capture of the most adverse background noise scenario as it records the maximum noise values. Consequently, any significant variation in the frequency spectrum can be attributed to the potential presence of suspicious partial discharge (PD) activity. Furthermore, this procedure was repeated for each set of equipment monitored at the substation, given that background noise may exhibit variations between successive measurements over time. The second step consisted of directing each antenna towards the equipment under analysis (PTs), as shown in Figure 3, in order to verify if there were significant differences between the extracted spectrum for the equipment and the reference one (background noise). Again, the frequency spectrum values were recorded via the spectrum analyzer after checking that they had stabilized.

From the values obtained using both the bio-inspired PMA and the commercial antenna in each one of the stages aforementioned, it was possible to compare the frequency spectra corresponding to the background noise and the analyzed equipment. Significant differences between these frequency spectra could be considered possible PDs from the evaluated PT. In this field experiment, in order to avoid the detection of false positives, only differences greater than 10 dBm were considered significant for the identification of suspected frequencies of PD activity. When using a high threshold such as this, only the PD signals that are actually coming from the PT to which the PMA is closest are detected. Due to the distance between the PMA and the equipment in other phases, the PD signals from the other PTs will be significantly attenuated during their propagation path to the PMA and, consequently, will not have enough intensity to exceed the established threshold of 10 dBm.

### 3.3. Laboratory Measurements of Partial Discharges in Potential Transformer

**Figure 4:**
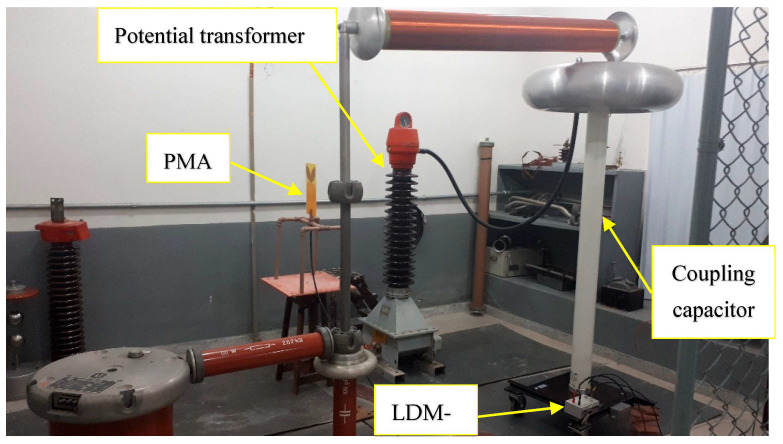
Laboratory experimental arrangement for PD measurement in the evaluated PT.

For the laboratory measurements, the experimental arrangement shown in Figure 4 was used, which involved a coupling capacitor (1000 pF) and a device for measuring impedance (LDM-5, from Doble Lemke, Dresden, Germany). The PT 82T3 phase A was subjected to simultaneous measurements using the bio-inspired PMA and the IEC 60270 method, with an LDS-6 digital measurement system from Doble Lemke. A Keysight oscilloscope DSO90604A with a bandwidth of 6 GHz, sampling rate of 20 GSa/s, rise time of 70 ps and four analog channels was connected to the bio-inspired PMA for the detection of PD signals in the time domain. Initially, with the arrangement de-energized, the background noise measurement was obtained using the LDS-6 and the bio-inspired PMA. After recording the background noise, the arrangement was energized at three voltage levels (80%, 100% and 115% of the nominal voltage of the potential transformer). For each applied voltage, PD pulses were captured using the bio-inspired PMA and the IEC 60270 method simultaneously.

**Figure 5:**
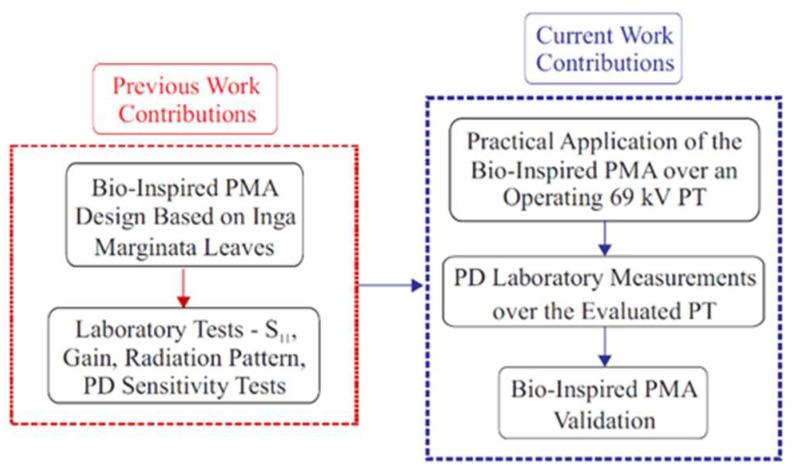
Summarized flowchart of the developed methodology.

In Figure 5, a summarized flowchart of the methodology developed in this work is presented, highlighting the differences between the contributions given in the previous work [22] and in the current work presented in this paper.

## 4. Results and Discussion

The presentation of the results obtained by the application of the proposed methodology is divided into two subsections. The first subsection is regarding the onsite (operating substation) PD measurement over the examined PT using the evaluated bio-inspired PMA; meanwhile, the second subsection is regarding the validation, in the laboratory (offline) and using the IEC 60270 method, of the results obtained at the first subsection.

### 4.1. Onsite Evaluation

**Figure 6:**
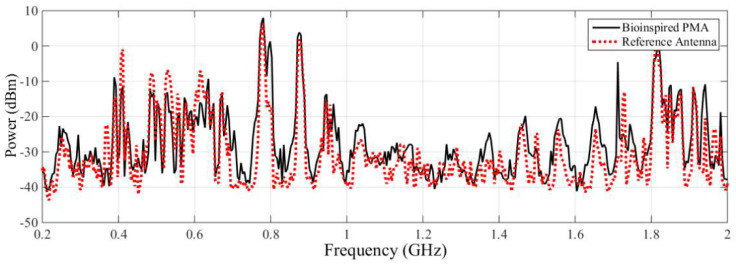
Comparison of the registered background noise between each antenna used (bio-inspired and commercial).

Figure 6 presents the background noise measurements obtained with the bio-inspired PMA and the commercial antenna. It can be seen that the background noise detected by each antenna shows similar frequencies, with small differences in amplitude due to the distinctive and characteristic gains of each antenna.

**Figure 7:**
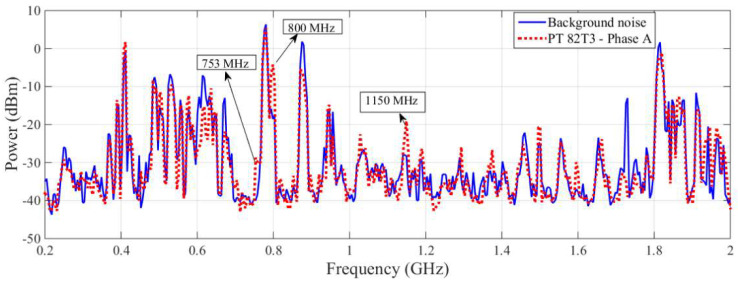
Spectral signature obtained by the commercial antenna for the PT 82T3 phase A.

**Figure 8:**
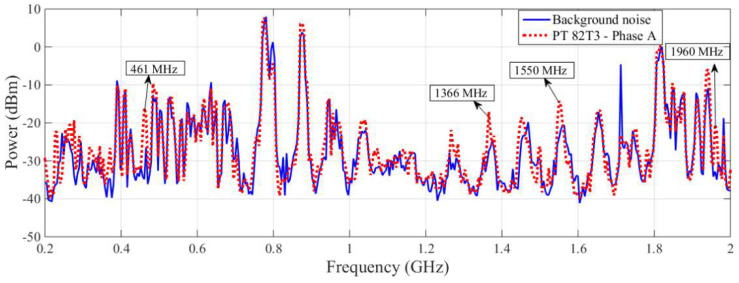
Spectral signature obtained by the bio-inspired PMA for the PT 82T3 phase A.

The spectral signatures detected by the commercial antenna and the bio-inspired PMA for the PT 82T3 phase A are shown in Figure 7 and Figure 8, respectively.

Despite the significant variation in power detected by the commercial antenna at frequencies of 753 MHz and 800 MHz, these frequencies were not considered to be suspicious for PD, since these frequencies showed similar power values to the bio-inspired PMA at the background noise extraction stage, as can be seen in Figure 6. The marked disparity in background noise levels between the two antennas at these two frequencies can be attributed to the distinctive radiation patterns of the employed antennas. As elucidated in [22], the radiation pattern of the bio-inspired antenna closely approximates an omnidirectional configuration, capturing background noise signals from all directions within the substation. Conversely, the commercial antenna features a highly directional radiation pattern, resulting in the acquisition of characteristic background noise signals from a specific direction. Consequently, when applying the proposed methodology for equipment monitoring, the directional nature of the commercial antenna may lead to the emergence of frequencies that could be erroneously interpreted as false positives. Operators might mistake background noise for partial discharges, as illustrated in Figure 7. The utilization of the developed bio-inspired antenna significantly diminishes the likelihood of false positives, as its omnidirectional pattern ensures that, regardless of the antenna’s orientation during background noise extraction, the noise background consistently yields similar results. This represents a notable advantage of employing the developed antenna in practical field applications as opposed to commercial directional antennas, as further exemplified in other cases discussed later in this study. However, based on the significant differences in the values for power, it is clear that the commercial antenna detected PD activity at a frequency of 1150 MHz.

**Figure 9:**
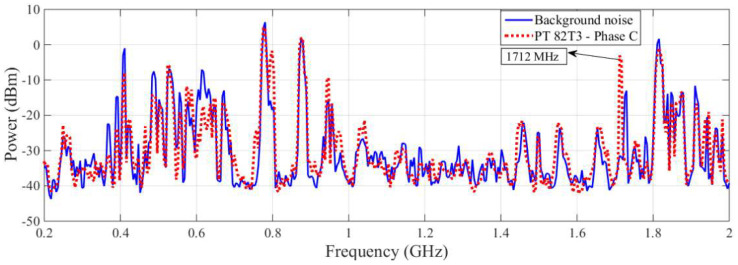
Spectral signature obtained by the commercial antenna for the PT 82T3 phase C.

**Figure 10:**
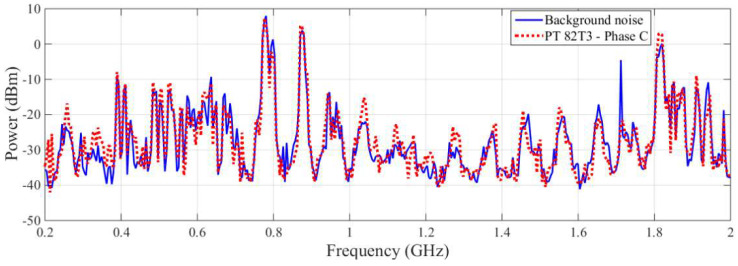
Spectral signature obtained by the bio-inspired PMA for the PT 82T3 phase C.

As expected, the results obtained during the laboratory tests in [22] show that the bio-inspired PMA showed greater detection sensitivity than the commercial antenna, since this bio-inspired PMA was designed specifically for the purpose of PD detection. By observing the spectral signature obtained for the PT 82T3 phase A, it can be seen that the PD activity can be verified more effectively with the bio-inspired PMA, as shown in Figure 8, and PD activity is highlighted at frequencies of 461, 1366, 1550 and 1960 MHz. The measurements for PT 82T3 phase B showed levels of PD similar to those for PT 82T3 phase A; hence, for greater objectivity in the presentation of this paper, only the results of the PT 82T3 phase A and phase C were selected. The spectral signatures detected by the commercial antenna and bio-inspired PMA for the PT 82T3 phase C are presented in Figure 9 and Figure 10, respectively.

Despite the detection by the commercial antenna of a significant variation in power at a frequency of 1712 MHz, as shown in Figure 9, the results for this frequency should not be considered a signal of PD, since the same frequency was detected by the bio-inspired PMA during the extraction of background noise. In addition, the other variations in power verified for the rest of the spectral signature for the commercial antenna were not significant, i.e., were lower than 10 dBm.

Even with the greater variation in power in comparison to the commercial antenna, as shown in Figure 10, the difference between the spectral signatures for the frequency range analyzed by the bio-inspired PMA was lower than 10 dBm. Hence, both antennas indicated a low possibility of PD activity for PT 82T3 phase C, and the presence of PD in PT 82T3 phase C was therefore excluded.

### 4.2. Offline (Laboratory) Validation Using the IEC 60270 Method

In order to verify the presence of PD after the analysis of the PT at the Mussuré II substation, the PT 82T3 phase A was collected for a more detailed inspection at the High Voltage Laboratory of UFCG, since experiments could be carried out in a controlled environment with less susceptibility to interference. PD measurements were performed using the bio-inspired PMA and the IEC 60270 standard method, using the LDS-6 digital measurement system and the experimental setup illustrated in Figure 4.

**Figure 11:**
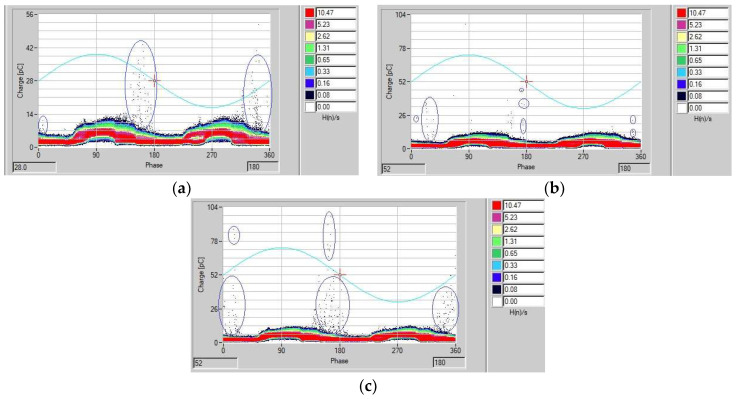
PRPD analysis: (**a**) 31.8 kV (80% of the phase-neutral voltage); (**b**) 39.8 kV (phase-neutral voltage) and (**c**) 45.8 kV (115% of the phase-neutral voltage).

With a phase-neutral voltage of 69/√3 kV, the PT was subjected to voltages below and above the operating voltage, at levels of 80% and 115%, respectively. Figure 11 presents the results obtained from the LDS-6, as represented in a phase-resolved partial discharge (PRPD) analysis, which consists of a graph of the apparent charge as a function of the phase angle for the PT 82T3 phase A. In this experiment, the evaluated PT showed PD activity above a voltage of 31.8 kV, equivalent to 80% of the operating voltage. Notice that the PD points highlighted by blue circles appear at the phase transitions, a common feature of PD activity [23]. Each recorded point inside the blue circles shown in Figure 11 represents the magnitude of the apparent charge (pC) of a single PD pulse. According to Ref. [27], in this type of PT, levels of PD of up to 10 pC are allowed. The results obtained here represent levels higher than allowed, with values of above 20 pC, thus proving that the analyzed PT does in fact show a high level of PD activity. The points that make up the red, yellow, green and blue bands (with values of around 10 pC) represent the background noise levels of the measurement environment and were previously identified with a de-energized arrangement in order to avoid detecting false positives.

**Figure 12:**
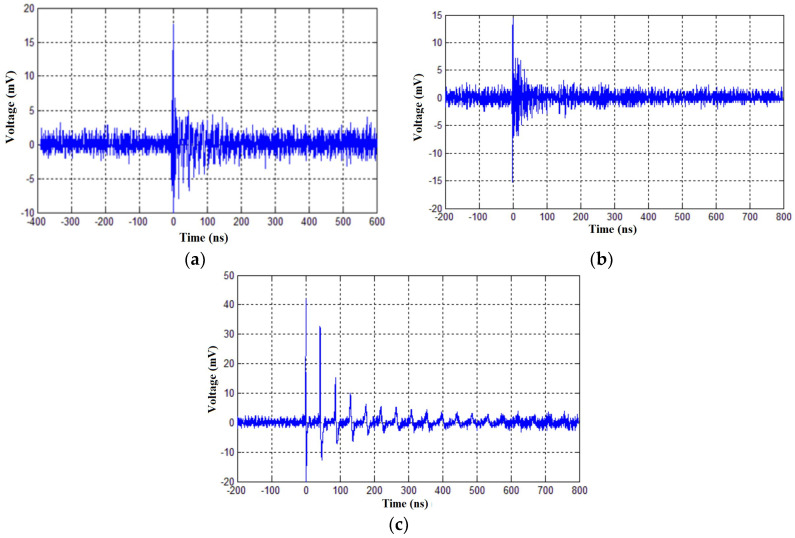
Partial discharge pulses detected in PT 82T3 phase A by the bio-inspired PMA: (**a**) 31.8 kV (80% of the phase-neutral voltage); (**b**) 39.8 kV (phase-neutral voltage) and (**c**) 45.8 kV (115% of the phase-neutral voltage).

Figure 12 shows pulses of PD in the time domain, detected by the bio-inspired PMA and the IEC 60270 method simultaneously, for voltage applications of 31.8, 39.8 and 45.8 kV to the evaluated PT.

It can be seen from Figure 11c that, for a voltage of 115% of the nominal voltage value, there is a significant partial discharge activity. This elevation may have had a direct impact on the formation mechanism of partial discharges internal to the equipment, resulting in changes in the shape of the observed pulse and, consequently, in the frequency of the UHF pulse detected by the bio-inspired antenna, as can be seen in Figure 12c. This phenomenon is mainly due to the stochastic nature of partial discharges; therefore, each partial discharge pulse is different from the other and may have slightly different frequency ranges and shapes within the UHF spectrum.

**Figure 13:**
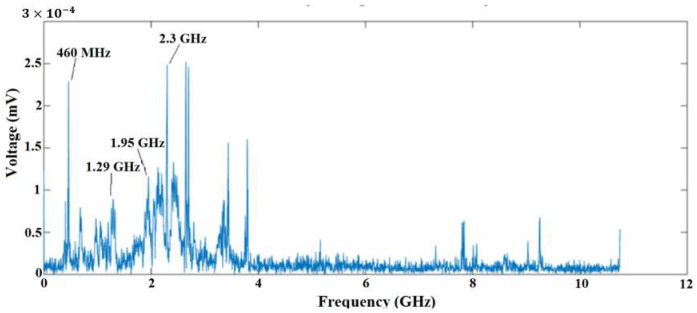
Partial discharge pulse measured by the bio-inspired PMA in the frequency domain.

To compare the results obtained at the substation with those obtained in the laboratory, Figure 13 shows the frequency spectrum of one of the PD pulses detected in the time domain for the nominal voltage. Notice that the frequency spectrum obtained in this experiment coincides with the values of frequency obtained from the tests at the substation for the same equipment (PT 82T3 phase A), mainly at a frequency of 460 MHz. This therefore confirms that the spectral signature identified for this PT via the measurements at the substation did in fact originate from PD activity.

## 5. Conclusions

In this work, we evaluated the practical application of a bio-inspired PMA, based on Inga Marginata leaves, for the detection of PDs in HV equipment operating in a substation.

The conclusions that can be drawn from the results of this paper can be summarized as follows:

- The measurements made at the substation identified possible frequencies of PD activity in two of the three 69 kV potential transformers (phases A and B), mainly at frequencies of 461, 1366, 1550 and 1960 MHz;
- The bio-inspired PMA had a higher sensitivity in terms of PD detection than the commercial antenna, and identified a larger number of possible frequencies of PD activity;
- During laboratory testing using the IEC 60270 standard method, the suspected presence of PD activity in the PT recorded at the substation was confirmed. The detected PD involved apparent charge values of above 20 pC, higher than the 10 pC limit established for this type of HV equipment;
- The bio-inspired PMA was able to detect PD activity for all voltage levels used (0.8 VN, VN and 1.15 VN) during laboratory testing, thus demonstrating its sensitivity in terms of PD detection in comparison with the IEC 60270 standard method, even for discharges with low intensity (20 pC);
- The frequency spectrum obtained from the PD signals detected by the bio-inspired PMA in the laboratory was similar to those obtained via the practical application in the substation, with a higher energy concentration at a frequency of 461 MHz, making it a possible indicator that can be used for PD classification through artificial intelligence.

An analysis of the PT both at the substation and in the laboratory proved the presence of PD. Hence, the experiments and results presented here verified the efficiency of the bio-inspired PMA based on Inga Marginata leaves, in terms of the practical detection of PD in HV equipment at an operating substation. Our bio-inspired PMA showed very promising results compared to established methods of detection such as IEC 60270, presenting good potential for future integration with commercial PD measurement systems that uses the radiometric method for detection and classification of PD through the application of artificial intelligence.

## Data Availability

The data presented in this study are available on request from the corresponding author. The data are not publicly available due to company privacy.
